# Supplementary material for: The impact of tax accounting and planning on earnings management: Evidence from panel ARDL approach
Source: PLoS One. 2025 Nov 14;20(11):e0336911. doi: 10.1371/journal.pone.0336911 (PMC12617935; doi:10.1371/journal.pone.0336911)
Supplement: Attachment — Submitted filename: Response_to_Reviewers.docx [file pone.0336911.s002.docx]

I am deeply grateful to the academic editor and reviewers for their insightful recommendations, which have helped refine this manuscript. I have carefully addressed all comments and implemented the suggested revisions to improve the quality of my manuscript. The revisions made taking into account the reviewers' comments are shown in red in the text and are listed below:

Rewiever 1:

I would like to extend my sincere gratitude to Reviewer 1 for their insightful comments and constructive feedback, as well as for their supportive and encouraging approach.

Rewiever 2:

Comment 1:

The theoretical rationale linking this indicator to tax planning is elaborated in the section pertaining to Equation 5.

Comment 2:

Robustness checks were conducted by incorporating firm size and leverage ratio as control variables, and the results are presented in the text.

Comment 3:

The procedure used to select the appropriate lag length are explained in the manuscript.

Comment 4:

The text explains how the presence and strength of cross-sectional dependence are accounted for in the PMG estimation framework.

Comment 5:

The keyword has been changed as suggested.

Comment 6:

The reference formatting has been revised to conform to the journal's style guidelines.

Comment 7:

The inclusion of new and recommended sources has served to strengthen the theoretical framework and literature review, thereby providing a more comprehensive academic foundation for the study.
